# Global small-angle X-ray scattering data analysis for multilamellar vesicles: the evolution of the scattering density profile model

**DOI:** 10.1107/S1600576713029798

**Published:** 2013-12-25

**Authors:** Peter Heftberger, Benjamin Kollmitzer, Frederick A. Heberle, Jianjun Pan, Michael Rappolt, Heinz Amenitsch, Norbert Kučerka, John Katsaras, Georg Pabst

**Affiliations:** a Instiute of Molecular Biosciences, Biophysics Division, University of Graz, Austria; b Biology and Soft Matter Division, Oak Ridge National Laboratory, Oak Ridge, TN, USA; c Department of Physics, University of South Florida, Tampa, FL 33620, USA; d Institute of Inorganic Chemistry, Graz University of Technology, Austria; e School of Food Science and Nutrition, University of Leeds, UK; f Canadian Neutron Beam Centre, National Research Council, Chalk River, ON, Canada; g Joint Institute for Neutron Sciences, Oak Ridge, TN, USA; h Department of Physics and Astronomy, University of Tennessee, Knoxville, TN, USA; i Department of Physics, Brock University, St Catharines, ON, Canada

**Keywords:** small-angle X-ray scattering, scattering density profile model, genetic algorithms, liquid crystalline multilamellar vesicles

## Abstract

The highly successful scattering density profile (SDP) model, used to jointly analyze small-angle X-ray and neutron scattering data from unilamellar vesicles, has been adapted for use with data from fully hydrated, liquid crystalline multilamellar vesicles (MLVs). Using a genetic algorithm, this new method is capable of providing high-resolution structural information, as well as determining bilayer elastic bending fluctuations from standalone X-ray data. Structural parameters such as bilayer thickness and area per lipid were determined for a series of saturated and unsaturated lipids, as well as binary mixtures with cholesterol. The results are in good agreement with previously reported SDP data, which used both neutron and X-ray data. The inclusion of deuterated and non-deuterated MLV neutron data in the analysis improved the lipid backbone information but did not improve, within experimental error, the structural data regarding bilayer thickness and area per lipid.

## Abstract 2

The high-resolution scattering length density analysis method has been generalized from unilamellar to multilamellar lipid vesicles. The method may be applied to X-ray data only, or to a joint analysis of X-ray and neutron data, yielding an improved refinement of the lipid backbone position.

## 1. Introduction   

Phospholipids are a major component of biological membranes, and the structural analysis of pure lipid membranes is an important area of research, as it can provide valuable insights into membrane function, including how the membrane’s mechanical properties affect lipid/protein interactions (Escribá *et al.*, 2008 ▶; Mouritsen, 2005 ▶). Of the liquid crystalline mesophases formed by phospholipids in aqueous solutions, most effort has been expended in studying fluid bilayers (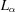), because of their commonly accepted biological significance.

Over the years, scattering techniques such as small-angle X-ray and neutron scattering (SAXS and SANS) have been widely used to determine the structural parameters and mechanical properties of biomimetic membranes. With regard to bilayer structure, two important structural parameters are bilayer thickness and lateral area per lipid *A* (Lee, 2004 ▶; Pabst *et al.*, 2010 ▶; Heberle *et al.*, 2012 ▶); the latter is directly related to lipid volume and inversely proportional to bilayer thickness. Importantly, *A* plays a key role in the validation of molecular dynamics (MD) simulations (Klauda *et al.*, 2006 ▶), and as such, its value for different lipids must be accurately known. Historically, for a given lipid, a range of values for *A* have been reported (Kučerka *et al.*, 2007 ▶). Since lipid volumes are determined from independent and highly accurate densitometry measurements (Nagle & Tristram-Nagle, 2000 ▶; Greenwood *et al.*, 2006 ▶; Uhríková *et al.*, 2007 ▶), differences in *A* must therefore result from differences in bilayer thickness. To accurately determine lipid areas, a precise measure of the Luzzati thickness 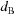 (Luzzati & Husson, 1962 ▶), which is given by the Gibbs dividing surface of the water/bilayer interface (Kučerka, Nagle *et al.*, 2008 ▶), is needed. Other frequently used definitions of bilayer thickness are the headgroup-to-headgroup thickness 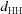 and the steric bilayer thickness (Pabst, Katsara *et al.*, 2003 ▶). The latter two bilayer thicknesses can also be used to determine *A*; however, assumptions regarding the headgroup size or the distance to the chain/headgroup interface have to be made.

There are two important issues that one must consider when measuring membrane thickness. Firstly, owing to the thermal disorder of fluid bilayers, there is no distinct division between lipids and water; instead a water concentration gradient exists at the membrane’s interface. Secondly, X-rays and neutrons are sensitive to different parts of the bilayer. X-rays, for example, are strongly scattered from the electron-dense phosphate group, which is part of the phosphorylcholine headgroup, and hence accurate values for 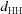 can be obtained. On the other hand, neutrons are scattered by atomic nuclei and can be used for contrast variation analyses, since hydrogen and its isotope deuterium scatter neutrons with similar efficiencies but 180° out-of-phase with each other (*i.e.* deuterium’s coherent scattering length is positive, while hydrogen’s is negative). In the case of protiated lipid bilayers, SANS is highly sensitive to locating the hydrogen-depleted carbonyl groups. Importantly, however, neutron contrast can be easily tuned by varying the hydrogen–deuterium content of the water (by varying the 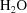/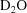 ratio) or of the bilayer (through the use of deuterated lipids) (Pabst *et al.*, 2010 ▶). As mentioned, in the case of protiated lipid bilayers in 100% 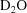, neutrons are most sensitive to the lipid’s glycerol backbone. Moreover, the Gibbs dividing surface for the apolar/polar interface is typically located between the headgroup phosphate and the lipid backbone. Therefore a combined analysis of X-ray and neutron data should yield the most accurate values of 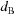 and *A* (Kučerka, Nagle *et al.*, 2008 ▶; Kučerka *et al.*, 2011 ▶; Pan, Heberle *et al.*, 2012 ▶). In this combined data analysis, commonly known as the scattering density profile (SDP) model, the lipid bilayer is represented by volume distributions of quasi-molecular fragments, which are easily converted into electron density or neutron scattering length density distributions by scaling them (for a given mol­ecular group) with the appropriate electron or neutron scattering length density [see Heberle *et al.* (2012 ▶) for a recent review].

Scattering techniques are also capable of probing membrane elasticity. Lipid bilayers are two-dimensional fluids which exhibit significant bending fluctuations of entropic origin. In multilamellar arrangements, *e.g.* in liquid crystalline multilamellar vesicles (MLVs) or surface-supported multibilayers, this leads to a characteristic power-law decay of the positional correlation function, known as quasi-long-range order, with Bragg peaks having characteristic line shapes (Liu & Nagle, 2004 ▶; Salditt, 2005 ▶; Pabst *et al.*, 2010 ▶). Membrane elasticity can therefore be determined from line-shape analysis of the Bragg peaks, and the underlying physics of this phenomenon is described by the Caillé (1972 ▶) or modified Caillé theory (MCT) (Zhang *et al.*, 1994 ▶). The resulting fluctuation, or Caillé parameter η, is a function of the bilayer bending modulus and the bulk modulus of interbilayer compression. Owing to the higher-resolution data, compared to neutrons, X-rays are better suited for line-shape analysis of Bragg peaks.

Just over a decade ago, Pabst and co-workers were the first to report a full-*q*-range analysis of MLV SAXS data using MCT (Pabst *et al.*, 2000 ▶; Pabst, Koschuch *et al.*, 2003 ▶). In that method, quasi-Bragg peaks and diffuse scattering were both taken into account when analyzing the data, and the electron density profile was modeled by a simple summation of Gaussians representing the electron-rich lipid headgroup and electron-poor (in relation to the headgroup) hydrocarbon chains. Selected examples of this SAXS method of data analy-sis can be found in the recent reviews by Pabst *et al.* (2010 ▶, 2012 ▶).

The work described here extends the global analysis program (GAP; Pabst *et al.*, 2000 ▶; Pabst, Koschuch *et al.*, 2003 ▶) for MLVs, by making use of the SDP description of the lipid bilayer. This modified technique, termed herein the SDP–GAP model, has several advantages. Firstly, compared to extruded unilamellar vesicles (ULVs), spontaneously forming MLVs are easier to prepare (Heberle *et al.*, 2012 ▶). Secondly, the SDP description of the bilayer imparts to GAP the ability to simultaneously analyze SANS and SAXS data, while enabling the SDP model to determine bending fluctuations and, hence, bilayer interactions.

In the present study we also attempted to determine precise values of 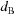 and *A* using standalone X-ray data. Such analysis, however, is complicated by the use of an increased number of fitting parameters, as compared to GAP, and inherently less scattering contrast, as compared to the SDP model, which simultaneously makes use of SANS and SAXS data. To address these shortcomings we used a genetic algorithm, as an optimization routine, in combination with information from other sources, thereby reducing the number of parameters needed by the SDP–GAP model. To test the new SDP–GAP model, we analyzed a series of saturated and unsaturated phospholipids, as well as binary lipid mixtures with cholesterol. The results compare favorably with previously reported data obtained using the SDP model, including the commonly accepted bilayer condensation effect induced by cholesterol. We also include SANS data of protiated and deuterated palmitoyl-oleoyl phosphatidylcholine (POPC) in our analysis, which gives rise to a better resolved location of the lipid’s glycerol backbone. Compared to standalone SAXS analysis, any differences in the values of *A* and 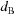 obtained from SDP-GAP model analysis are well within experimental uncertainty.

## 2. Material and methods   

### 2.1. Sample preparation   

1,2-Dipalmitoyl-*sn*-*glycero*-3-phosphocholine (DPPC), 1-pal­mitoyl-2-oleoyl-*sn*-*glycero*-3-phosphocholine (POPC), 1-pal­mit­oyl(d31)-2-oleoyl-*sn*-*glycero*-3-phosphocholine (POPC-d31), 1-stearoyl-2-oleoyl-*sn*-*glycero*-3-phosphocholine (SOPC) and 1,2-di­oleoyl-*sn*-*glycero*-3-phosphocholine (DOPC) were purchased from Avanti Polar Lipids, Alabaster, AL, USA, and cholesterol was obtained from Sigma–Aldrich (Austria). 99.8% 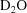 was obtained from Alfa Aesar (Ward Hill, MA, USA). All lipids were used without further purification.

For X-ray experiments, lipid stock solutions (DPPC, POPC, SOPC, DOPC) were prepared by dissolving predetermined amounts of dry lipids in chloroform/methanol (2:1, *v*/*v*). Binary mixtures with cholesterol (20 mol%) were obtained by mixing lipid stock solutions in the appropriate ratios. Lipid solutions were subsequently dried under a stream of nitrogen and placed under vacuum for about 12 h, forming a thin lipid film on the bottom of glass vials. Films were hydrated using 18 MΩ cm^−1^ water by incubation for 2 h above the lipid melting temperature, with vortex mixing every 15 min. The final lipid concentration for each sample was 50 mg ml^−1^.

For neutron experiments, MLVs of POPC-d31 at 10 mg ml^−1^ were prepared by weighing 15 mg of dry lipid powder into 13 × 100 mm glass culture tubes and hydrating with 1.50 ml 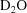 preheated to 313 K, followed by vigorous vortexing to disperse the lipid. The resultant MLV suspension was incubated at 313 K for 1 h, with intermittent vortexing, and then subjected to five freeze/thaw cycles between 193 and 313 K to reduce the average number of lamellae and facilitate extrusion (Kaasgaard *et al.*, 2003 ▶; Mayer *et al.*, 1985 ▶). A 0.75 ml aliquot of the MLV sample was used to prepare ULVs using a hand-held miniextruder (Avanti Polar Lipids, Alabaster, AL, USA) assembled with a 50 nm-pore-diameter polycarbonate filter and heated to 313 K. The suspension was passed through the filter 41 times. ULV samples were measured within 24 h of extrusion. The final sample concentrations were 10 mg ml^−1^, which allows for sufficient water between vesicles to eliminate the interparticle structure factor, thereby simplifying data analysis.

### 2.2. Small-angle X-ray scattering   

X-ray scattering data were acquired at the Austrian SAXS beamline, which is situated at the Elettra synchrotron (Trieste, Italy), using 8 keV photons. Diffraction profiles were detected utilizing a Mar300 image-plate detector (Marresearch GmbH, Norderstedt, Germany) and calibrated using a powder sample of silver behenate. Lipid dispersions were taken up in 1 mm-thick quartz capillaries and inserted into a multi-position sample holder. Samples were equilibrated for a minimum of 10 min prior to measurement at a predetermined temperature with an uncertainty of 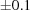
 K using a circulating water bath. The exposure time was set to 240 s. Scattering patterns were integrated using the program *FIT2D* (Hammersley, 1997 ▶). Background scattering originating from water and air was subtracted, and data sets were normalized using the transmitted intensity, which was measured by a photodiode placed in the beamstop.

### 2.3. Small-angle neutron scattering   

Neutron scattering experiments were performed using the Extended-*Q*-range Small-Angle Neutron Scattering (EQ-SANS, BL-6) instrument at the Spallation Neutron Source (SNS) located at Oak Ridge National Laboratory (ORNL). ULVs were loaded into 2 mm-path-length quartz banjo cells (Hellma USA, Plainview, NY, USA) and mounted in a temperature-controlled cell paddle with a 1 K accuracy. In 60 Hz operation mode, a 4 m sample-to-detector distance with a 2.5–6.1 Å wavelength band was used to obtain the relevant wavevector transfer. Scattered neutrons were collected with a two-dimensional (1 × 1 m) 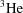 position-sensitive detector made up of 192 × 256 pixels. Two-dimensional data were reduced using *MantidPlot* (http://www.mantidproject.org/). During data reduction, the measured scattering intensity was corrected for detector pixel sensitivity, dark current, sample transmission, and background scattering contribution from the water and empty cell. The one-dimensional scattering intensity, *I versus q*, was obtained by radial averaging of the corrected two-dimensional data.

### 2.4. Modeling of phospholipid bilayer   

To analyze the scattering profile of MLVs, we adopted the full-*q*-range GAP model of Pabst and co-workers (Pabst *et al.*, 2000 ▶; Pabst, Koschuch *et al.*, 2003 ▶), which takes into account diffuse scattering *N*_diff_ originating from positionally uncorrelated bilayers):
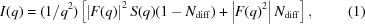
where the scattering vector magnitude 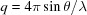, λ is the wavelength, 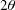 is the scattering angle relative to the incident beam, 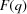 is the bilayer form factor and 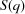 is the inter-bilayer structure factor. For fluid lipid bilayers, 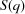 is given by the Caillé theory, which is described in detail elsewhere (Caillé, 1972 ▶; Zhang *et al.*, 1994 ▶; Pabst *et al.*, 2000 ▶; Pabst, Koschuch *et al.*, 2003 ▶). Averaging over variations in scattering domain size was performed following Frühwirth *et al.* (2004 ▶). One of the important parameters determined from fitting 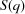 using MCT is the Caillé parameter η, which is a measure of bending fluctuations (Pabst *et al.*, 2010 ▶). The number of positionally correlated bilayers 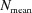 affects the width of the Bragg peaks (Pabst, Koschuch *et al.*, 2003 ▶) and must be optimized through fitting of the data. In the case of the present samples, the number of bilayers 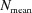 contributing to Bragg scattering varied between ten and 30. Instrumental resolution was taken into account by convoluting equation (1) with the beam profile (Pabst *et al.*, 2000 ▶; Qian & Heller, 2011 ▶), and incoherent background scattering was accounted for by an additive constant.

**Figure 1:**
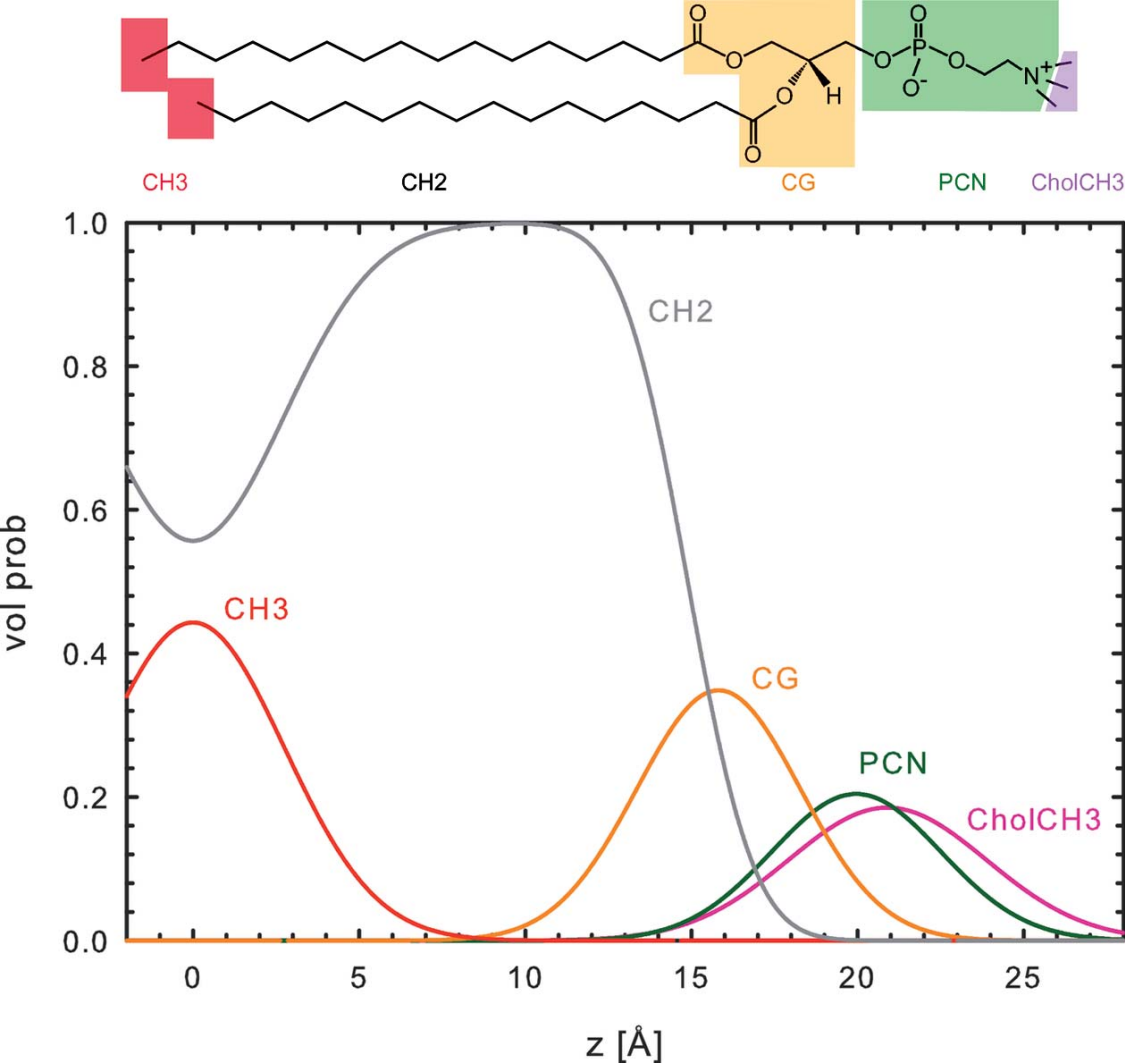
Illustration of the bilayer parsing scheme (top panel) and volume probability distribution (bottom panel) for DPPC. Data are from experiments carried out in the present study.

The form factor is the Fourier transform of the electron density or neutron scattering length density profile. In the present study, we implemented the SDP model (Kučerka, Nagle *et al.*, 2008 ▶) to describe the bilayer. The SDP model describes the membrane in terms of the volume distributions of quasi-molecular fragments. A detailed description of volume probability distribution functions is given in the article by Kučerka, Nagle *et al.* (2008 ▶). The water-subtracted scattering length density distributions [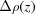] are calculated by scaling the volume probability distributions using component total electron densities (for X-rays) or neutron scattering length densities. The form factor is then calculated as
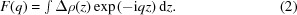
Kučerka and co-workers originally parsed phosphatidylcholines into the following components: choline methyl (CholCH_3_); phosphate + CH_2_CH_2_N (PCN); carbonyl + glycerol (CG); hydrocarbon methylene (CH_2_); and hydrocarbon terminal methyl (CH_3_). An additional methine (CH) group was added for unsaturated hydrocarbon chains. However, the contrast between CH and CH_2_ is weak, even for SANS (Kučerka, Nagle *et al.*, 2008 ▶), and effectively zero for SAXS. Hence, our parsing scheme combined the CH with the CH_2_ group (Fig. 1 ▶).

To avoid nonphysical results, the following constraints were adopted following Klauda *et al.* (2006 ▶) and Kučerka, Nagle *et al.* (2008 ▶). Because of bilayer symmetry, the position of the terminal methyl group 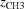 was set to zero and the height of the error function, which describes the hydrocarbon chains, was set to one in order to comply with spatial conservation. The width of the choline methyl group 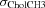 was fixed to 2.98 Å, and the width of the error function describing the hydrocarbon chain was constrained within accepted limits (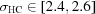
 Å) (Klauda *et al.*, 2006 ▶; Kučerka, Nagle *et al.*, 2008 ▶).

We also implemented new constraints to aid the standalone X-ray data analysis. Firstly, the distances between the CholCH_3_ and PCN groups, and the hydrocarbon chain interface (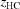) and CG (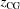) groups, were not allowed to exceed 2 Å because of their spatial proximity. Secondly, volumes of the quasi-molecular fragments, necessary for calculating electron or neutron scattering length densities, were taken from previous reports (Kučerka *et al.*, 2005 ▶, 2011 ▶; Kučerka, Nagle *et al.*, 2008 ▶; Klauda *et al.*, 2006 ▶; Greenwood *et al.*, 2006 ▶) and allowed to vary by 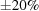. The total volume of the headgroup components (*i.e.* CholCH_3_, PCN and CG) was constrained to a target value of 331 Å^3^, as reported by Tristram-Nagle *et al.* (2002 ▶), whereby the value is allowed to deviate from the target value, but in doing so, incurs a goodness-of-fit penalty.

For lipid mixtures with cholesterol, cholesterol’s volume distribution was merged with that of the CH_2_ group, following Pan, Cheng *et al.* (2012 ▶). This is justified on the basis of cholesterol’s strong hydrophobic tendency, which dictates its location within the hydrocarbon chain region, and the fact that its hydroxy group resides in the vicinity of the apolar/polar interface (Pan, Cheng *et al.*, 2012 ▶). In calculating the lipid area for binary mixtures, the apparent area per lipid 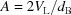 was used (Pan, Cheng *et al.*, 2012 ▶; Pan *et al.*, 2009 ▶). The volume of cholesterol within lipid bilayers was taken to be 630 Å^3^ (Greenwood *et al.*, 2006 ▶).

### 2.5. Determination of structural parameters   

On the basis of volume probability distributions and scattering length density profiles, membrane structural parameters were defined as follows: (i) the headgroup-to-headgroup distance 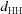 is the distance between maxima of the total electron density (*i.e.* the sum of the component distributions); (ii) the hydrocarbon chain length 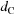 is the position of the error function representing the hydrocarbon region 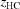; and (iii) the Luzzati thickness 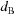 is calculated from the integrated water probability distribution (Kučerka, Nagle *et al.*, 2008 ▶):
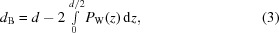
where *d* is the lamellar repeat distance. The volume distribution function of water was previously defined as (Kučerka, Nagle *et al.*, 2008 ▶)
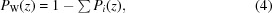
where *i* indexes the lipid component groups (*i.e.* CholCH_3_, PCN, CG, CH_2_ and CH_3_). In order to increase the robustness of the analysis for 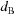, 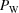 obtained from the SDP analysis was fitted with an error function, thus giving greater weight to the region close to the lipid headgroup (owing to the higher X-ray contrast) compared to the hydrocarbon chain region. We also attempted to include the 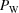 model function in the SDP fit; however, the results were not satisfactory. The area per lipid is then given by (Kučerka, Nagle *et al.*, 2008 ▶)
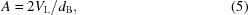
where 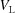 is the molecular lipid volume determined by separate experiments. Finally, the thickness of the water layer was defined as
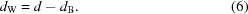



### 2.6. Fitting procedure   

Owing to the large number of adjustable parameters (*i.e.* 21) and our goal to apply the SDP–GAP model to standalone X-ray data, we chose to use a genetic algorithm in the optimization routine. The main benefit of this algorithm, compared to simple gradient descent routines or more sophisticated optimization algorithms (*e.g.* Levenberg–Marquardt), is that the fitting procedure does not easily fall into local minima (Goldberg, 1989 ▶). Briefly, a random set of adjustable parameters (termed a population) is chosen within fixed boundaries and tested for its fitness, defined here as the reduced chi squared (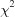) value, which is equal to the sum of the squared residuals divided by the degrees of freedom (Press *et al.*, 2007 ▶). The best solutions are then combined to obtain a new and better population, in a manner similar to the evolutionary process of genetic recombination [for details, see Goldberg (1989 ▶)]. Several hundred generations with populations of ∼2000 individuals were tested for their fitness. If 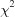 does not change after 100 generations, the optimization is assumed to have converged and the routine is terminated. Solutions with the lowest 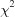 values are then compared with respect to differences in structural parameters. From the resulting distributions we estimate that the uncertainty of all parameters reported in the present work is 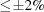. Application of genetic algorithms comes with a greater computational cost, and they are most efficient when using parallel processing techniques. For the present study, all routines were encoded in IDL (Interactive Data Language), using the *SOLBER* optimization routine (Rajpaul, 2012 ▶). Typical runtimes for one X-ray scattering profile were between three and five hours on a six core machine (Intel Xeon 2.67 GHz).

## 3. Results and discussion   

### 3.1. X-ray standalone data   

**Figure 2:**
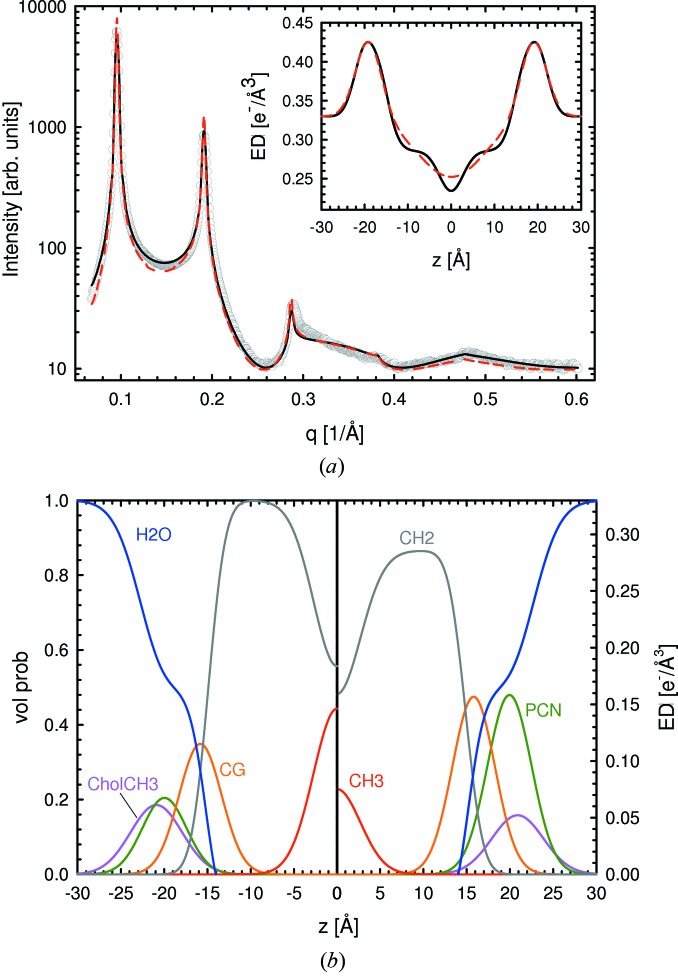
SDP–GAP analysis of SOPC MLVs at 303 K. Panel (*a*) compares the SDP–GAP (black line) and GAP models (red dashed line) with experimental data (grey circles). The inset to the figure compares the corresponding electron density profiles. Panel (*b*) shows the volume probability distribution (left hand side) and the electron density distributions of the defined quasi-molecular fragments (right hand side).

The SDP–GAP model was tested on SAXS data obtained from single component 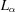 lipid bilayers and selected binary mixtures of phosphatidylcholines with cholesterol. As an example of our analysis, we present results for SOPC bilayers with five lamellar diffraction orders (Fig. 2 ▶). Fits from all other bilayers, including tables with structural parameters, are given in the supporting material (Figs. S1–S3, Table S1).^1^ All SAXS patterns showed significant diffuse scattering, originating from membrane fluctuations common to 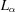 bilayers. In particular, bending fluctuations lead to a rapid decrease in diffraction peak amplitudes as a function of *q*, and quasi-Bragg peaks with characteristic line shapes. Such effects are accounted for in the structure factor used. We found good agreement between the SDP–GAP model and experimental SOPC data (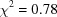). Fits from other MLV systems yielded similar 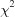 values (Table S1). Omitting the constraints introduced in §2.4 led to slightly improved 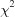 values but produced nonphysical results.

Results from the SDP–GAP model were compared with those from the GAP model. The GAP data were in reasonable agreement with the experimental data (Fig. 2 ▶), albeit with poorer fit statistics (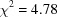), which could be attributed to the small deviations of the model between the various Bragg peaks. Despite the good fits produced using the GAP model, the structural features obtained from SDP–GAP analysis are significantly richer (Fig. 2 ▶, lower panel). This point is illustrated by the total electron density shown in the inset to Fig. 2 ▶, where the methyl trough is smeared out in the GAP electron density profile.

Table 1 ▶ provides the main structural parameters obtained from SDP–GAP and GAP analyses of the same data, as well as literature values obtained from SDP analysis (*i.e.* joint refinement of SAXS and SANS data). The calculation of structural parameters using the GAP model is detailed by Pabst, Katsaras *et al.* (2003 ▶). Our results using the SDP–GAP model are in good quantitative agreement with the reference data. Deviations from the GAP model are, however, larger (though still reasonable) because of the simplified electron density model that was used. Interestingly, in the case of some lipids, we also find significant differences for the fluctuation parameter; these are attributable to the form factor, which modulates peak intensity. It therefore stands to reason that the better fits to the experimental data by the SDP–GAP model should result in more accurate η values.

**Figure 3:**
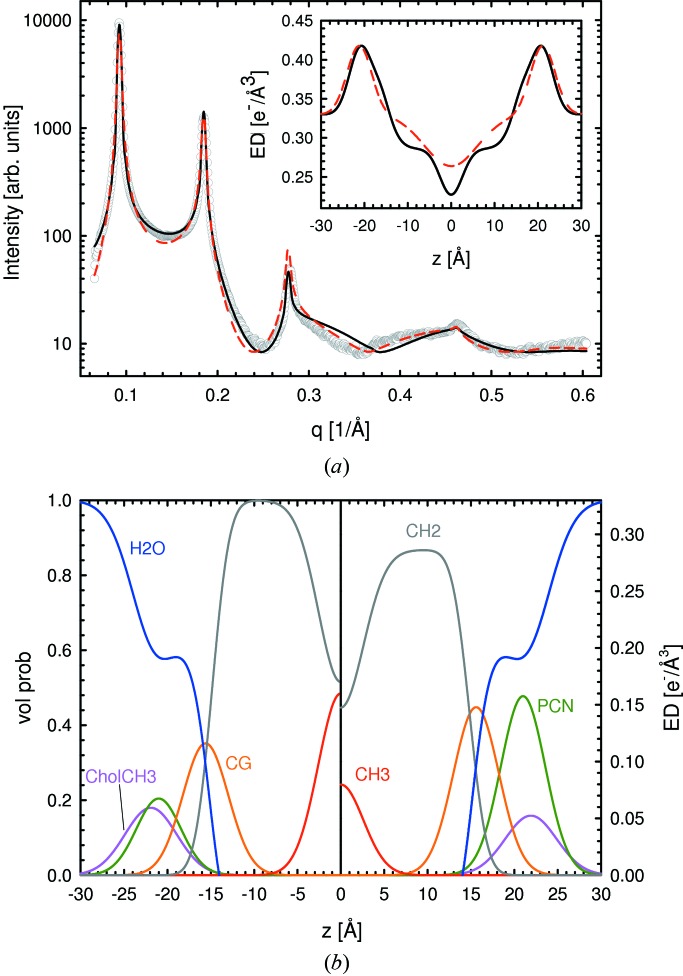
Comparing SDP–GAP and GAP fits to data from SOPC MLVs, with 20 mol% cholesterol at 303 K. The meaning of the lines is the same as in Fig. 2 ▶.

We further tested the SDP–GAP model using the same lipid systems, but this time with the addition of 20 mol% cholesterol. Cholesterol is abundant in mammalian plasma membranes and is well known for the condensing effect it has on lipid bilayers, which at the molecular level is explained by the umbrella model (Huang & Feigenson, 1999 ▶). In scattering studies, this effect shows up as an increase in 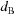 and a concomitant decrease in *A*, as well as in reduced bending fluctuations (see *e.g.* Hodzic *et al.*, 2008 ▶). Fig. 3 ▶ shows the fits to SOPC/cholesterol membrane data. The SDP–GAP model is able to describe the better resolved higher diffraction orders resulting from the presence of cholesterol. Our results show that cholesterol shifts the PCN and CholCH_3_ groups further away from the bilayer center (Fig. 3 ▶, bottom panel, and Tables 2 ▶ and S2), in good agreement with previous reports (Pan, Cheng *et al.*, 2012 ▶). On the other hand, we could not observe a significant shift of the CG group from the bilayer center or a higher value for the hydrocarbon chain thickness (Tables 2 ▶ and S2).

Structural parameters for all lipid mixtures are reported in Table 2 ▶. In agreement with previous reports, the addition of cholesterol causes *A* to decrease and 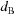 and 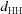 to increase (Hung *et al.*, 2007 ▶; Kučerka, Perlmutter *et al.*, 2008 ▶; Pan *et al.*, 2008 ▶; Hodzic *et al.*, 2008 ▶; Pan, Cheng *et al.*, 2012 ▶). Compared to other membrane systems, bending fluctuations in DPPC bilayers experience a greater degree of damping when cholesterol is introduced, in agreement with the notion that cholesterol preferentially associates with saturated hydrocarbon chains (Pan *et al.*, 2009 ▶, 2008 ▶; Ohvo-Rekilä *et al.*, 2002 ▶). This effect is smaller for lipids having one monounsaturated chain (*i.e.* SOPC and POPC) and is completely absent when a second monounsaturated chain is introduced (*e.g.* DOPC). This latter finding is in good agreement with studies that reported no change in the bending rigidity of DOPC bilayers in the absence or presence of cholesterol (Pan *et al.*, 2008 ▶).

SOPC/cholesterol mixtures were also analyzed with the GAP model. Although reasonable fits are obtained (Fig. 3 ▶, 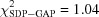, 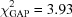), the differences in structural parameters when comparing GAP data with SDP–GAP data are more pronounced. For example, the total electron density profiles show clear deviations in the acyl chain and headgroup regions. Cholesterol increases the asymmetry of the electron density distribution in the headgroup region, as determined from the SDP–GAP model, an effect that is not captured by the single-headgroup Gaussian of the GAP model. As a result, parameters such as area per lipid (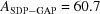
 Å^2^, 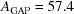
 Å^2^) and hydrocarbon chain length (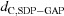 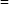 14.9 Å, 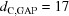
 Å) differ between the two methods, whereas the values for headgroup-to-headgroup thickness (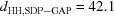
 Å, 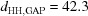
 Å) and the Caillé parameter (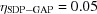, 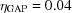) are in reasonably good agreement.

### 3.2. Addition of SANS data   

SANS data were obtained from POPC and POPC-d31 MLVs and ULVs in pure 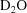 to see whether or not additional information substantially alters the results. The protocol devised by Kučerka and co-workers used SANS data from protiated bilayers at different 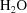/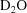 contrasts (Kučerka, Nagle *et al.*, 2008 ▶).

**Figure 4:**
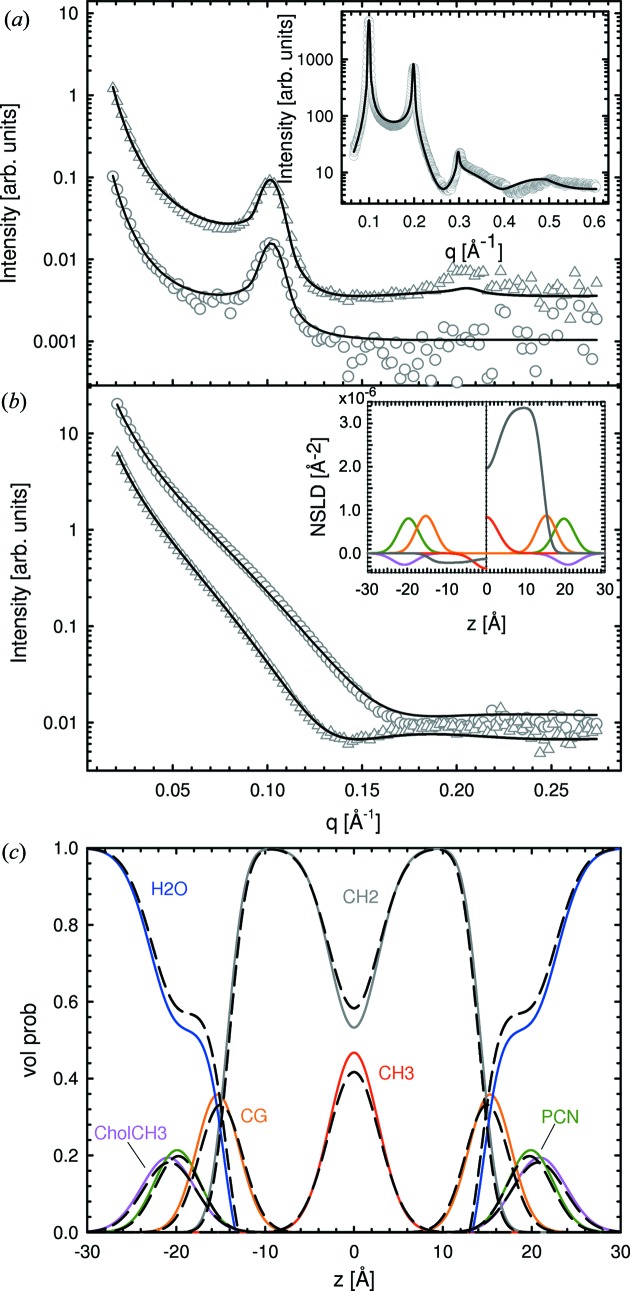
Results of simultaneous SAXS and SANS analysis of data from POPC ULVs and MLVs at 303 K. Panel (*a*) shows SANS data of POPC (circles) and POPC-d31 (triangles) MLVs, and corresponding data obtained from ULVs (same symbols) are shown in panel (*b*). Solid lines are best fits to the data using the SDP–GAP model. The insets in panels (*a*) and (*b*) show the corresponding SAXS fits and neutron scattering length density profiles for POPC (left) and POPC-d31 (right), respectively. Panel (*c*) shows the changes in volume distributions from a SAXS-only analysis (dashed black lines) to a simultaneous SAXS/SANS analysis (colored lines; same color coding as in Figs. 2 ▶ and 3 ▶).

Replacing H with D shifts the neutron scattering length density (NSLD) profile of the hydrocarbon region from negative to positive values (Fig. 4 ▶ *b*, inset). Hence, relative to 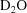 with an SLD = 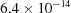
 cm Å^3^, the hydrocarbon chain region contrast is significantly altered. This change in contrast manifested itself by producing two additional Bragg peaks in the case of POPC-d31 MLVs, compared to their protiated counterparts (Fig. 4 ▶ *a*). Similarly, ULV data show a shift of the minimum at low *q* to higher *q* vector magnitudes for POPC compared to POPC-d31 (Fig. 4 ▶ *b*), which is also attributed to the change in contrast of the deuterated lipids in 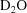.

We used SDP–GAP to simultaneously analyze SAXS data in several combinations with SANS data: (i) protiated MLVs; (ii) deuterated MLVs; and (iii) all four SANS data sets (*i.e.* deuterated and protiated MLVs and ULVs). We also fitted all MLV data sets simultaneously and all ULV data sets separately. Fit results are shown in Fig. 4 ▶ and the determined structural parameters are summarized in Tables 3 ▶ and S3. The addition of a single SANS data set produced variations in the structural parameters, causing them to deviate from values determined from standalone SAXS analysis and those from the literature. This disagreement was rectified by including either both MLV data sets or all MLV and ULV data sets in the analysis. In the latter case, significant differences, compared to the standalone SAXS analysis, are found regarding the positions of the CG group, 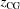 and 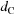. This can be understood in terms of the better neutron contrast of the lipid backbone. Changes in volume distribution functions are shown in Fig. 4 ▶(*c*). The changes to *A* and 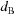 are within the experimental error and consequently of no significance. We thus conclude that the addition of SANS data helps to improve the location of the CG group and 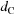, but offers little improvement to values of *A* and 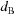.

## 4. Conclusion   

We have modified the full-*q*-range SAXS data analysis, which previously used a simplified electron density profile (Pabst *et al.*, 2000 ▶), with a high-resolution representation of scattering density profiles, based on volume distributions of quasi-molecular fragments (Kučerka, Nagle *et al.*, 2008 ▶). The new SDP–GAP method, as its name implies, is a hybrid model that combines advantages offered by the GAP and SDP models. The SDP–GAP model can be used to analyze MLV and ULV data and is capable of simultaneously analyzing SAXS and SANS data. MLVs are spontaneously formed membrane systems, and the development of this new hybrid model opens up new opportunities for the study of their bilayer interactions and membrane mechanical properties (*e.g.* elasticity) (Pabst *et al.*, 2010 ▶).

An additional feature of this new model is its ability to obtain high-resolution structural information from standalone SAXS data. This is achieved by implementing an optimization routine based on a genetic algorithm, which is able to deal with the large number of adjustable parameters, even though additional constraints and input parameters are required in order to limit parameter space. Compared to the GAP and SDP models, which use Levenberg–Marquardt and downhill simplex optimization routines, respectively, the computational effort required by the SDP–GAP model is significantly higher. Typical CPU times on parallel processors are of the order of a few hours, as compared to a few minutes for SDP or GAP. However, an advantage is that the genetic algorithm prevents the optimization routine from stalling in local minima. By using different seeds for the random number generator, robust results with good convergence are readily obtained

We have tested the SDP–GAP model using different saturated and unsaturated phosphatidylcholine bilayers, with and without cholesterol. Results for 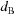 and *A* are in good agreement with previous reports using the SDP model, although we note that the position and width of the CG group are subject to greater variabilities, as a result of the lower X-ray contrast of this particular group. This inadequacy was, however, ameliorated by including ULV SANS data. MLV SAXS data combined with ULV SANS data of POPC and POPC-d31 bilayers improved both the position of the CG group and the hydrocarbon chain thickness (Fig. 3 ▶ *c* and Table 3 ▶). However, the values of *A* and 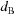 remained practically unchanged.

## Figures and Tables

**Table 1:** Comparison of structural parameters Parameter uncertainties are estimated to be 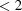
% as described in *Materials and methods* [Sec sec2].

		SDP–GAP	GAP	SDP†
DPPC (323 K)	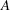 (Å^2^)	63.1	61.8	63.1
	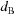 (Å)	39.0	n.a.	38.9
	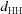 (Å)	37.9	37.3	38.4
	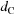 (Å)	13.9	14.5	14.2
	η	0.08	0.067	n.a.
POPC (303 K)	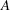 (Å^2^)	65.4	64.3	64.4
	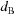 (Å)	38.4	n.a.	39.0
	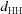 (Å)	37.3	37.0	36.5
	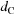 (Å)	14.0	14.4	14.4
	η	0.06	0.056	n.a.
SOPC (303 K)	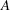 (Å^2^)	66.3	60.3	65.5
	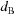 (Å)	39.5	n.a.	40.0
	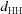 (Å)	38.7	40.7	38.6
	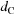 (Å)	14.6	16.2	15.0
	η	0.06	0.08	n.a.
DOPC (303 K)	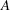 (Å^2^)	67.6	69.7	67.4
	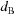 (Å)	38.5	n.a.	38.7
	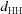 (Å)	36.9	36.1	36.7
	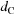 (Å)	14.2	13.9	14.4
	η	0.1	0.1	n.a.

† From Kučerka, Nagle *et al.* (2008 ▶) and Kučerka *et al.* (2011 ▶).

**Table 2:** Structural parameters from the SDP–GAP model of lipid bilayers containing 20 mol% cholesterol Parameter uncertainties are estimated to be 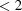
% as described in *Materials and methods* [Sec sec2].

Lipid	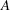 (Å^2^)	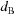 (Å)	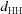 (Å)	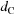 (Å)	η
DPPC (323 K)	61.2	40.1	42.3	14.2	0.02
POPC (303 K)	63.1	39.8	40.3	14.3	0.05
SOPC (303 K)	60.6	40.5	42.1 (42.1)†	14.9 (16.1)†	0.05
DOPC (303 K)	66.2	39.4	40.9 (39.0)†	13.5 (14.6)†	0.14

† From Pan *et al.* (2009 ▶).

**Table 3:** Structural parameters for POPC using different combinations of SAXS and SANS data Parameter uncertainties are estimated to be 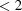
% as described in *Materials and methods* [Sec sec2].

	SAXS†	n-MLV 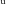 ‡	n-MLV 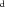 §	All data¶	SDP††
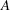 (Å^2^)	65.4	64.9	63.1	63.6	64.4
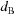 (Å)	38.4	38.7	39.8	39.5	39.0
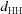 (Å)	37.3	37.1	37.3	37.5	36.5
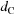 (Å)	14.0	14.6	14.4	14.3	14.4
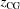 (Å)	15.0	15.3	15.4	15.3	15.3

† Results obtained using SAXS data only.

‡ SAXS (POPC–MLV) and SANS (POPC–MLV) data.

§ SAXS (POPC–MLV) and SANS (POPC-d31–MLV) data.

¶ SAXS (POPC–MLV) and SANS (POPC–ULV/MLV, POPC-d31–ULV/MLV) data.

†† From Kučerka *et al.* (2011 ▶).

## Footnotes

1 Supporting information for this article is available from the IUCr electronic archives (Reference: FS5056).
